# Biotransformation of Phthalate Plasticizers and Bisphenol A by Marine-Derived, Freshwater, and Terrestrial Fungi

**DOI:** 10.3389/fmicb.2020.00317

**Published:** 2020-02-28

**Authors:** Lena Carstens, Andrew R. Cowan, Bettina Seiwert, Dietmar Schlosser

**Affiliations:** ^1^Department of Environmental Microbiology, Helmholtz Centre for Environmental Research - UFZ, Leipzig, Germany; ^2^Institute for Environmental Microbiology and Biotechnology, University of Duisburg-Essen, Essen, Germany; ^3^Department of Analytical Chemistry, Helmholtz-Centre for Environmental Research - UFZ, Leipzig, Germany

**Keywords:** biosorption, biotransformation, cytochrome P450, endocrine disrupting chemicals, fungi, micropollutant, phthalate esters, plastic additives

## Abstract

Phthalate esters (PEs, Phthalates) are environmentally ubiquitous as a result of their extensive use as plasticizers and additives in diverse consumer products. Considerable concern relates to their reported xenoestrogenicity and consequently, microbial-based attenuation of environmental PE concentrations is of interest to combat harmful downstream effects. Fungal PE catabolism has received less attention than that by bacteria, and particularly fungi dwelling within aquatic environments remain largely overlooked in this respect. We have compared the biocatalytic and biosorptive removal rates of di-*n*-butyl phthalate (DBP) and diethyl phthalate (DEP), chosen to represent two environmentally prominent PEs of differing structure and hydrophobicity, by marine-, freshwater-, and terrestrial-derived fungal strains. Bisphenol A, both an extensively used plastic additive and prominent environmental xenoestrogen, was included as a reference compound due to its well-documented fungal degradation. Partial pathways of DBP metabolization by the ecophysiologically diverse asco- and basidiomycete strains tested were proposed with the help of UPLC-QTOF-MS analysis. Species specific biochemical reaction steps contributing to DBP metabolism were also observed. The involved reactions include initial cytochrome P450-dependent monohydroxylations of DBP with subsequent further oxidation of related metabolites, de-esterification via either hydrolytic cleavage or cytochrome P450-dependent oxidative *O*-dealkylation, transesterification, and demethylation steps - finally yielding phthalic acid as a central intermediate in all pathways. Due to the involvement of ecophysiologically and phylogenetically diverse filamentous and yeast-like fungi native to marine, freshwater, and terrestrial habitats the results of this study outline an environmentally ubiquitous pathway for the biocatalytic breakdown of plastic additives. Beyond previous research into fungal PE metabolism which emphasizes hydrolytic de-esterification as the primary catabolic step, a prominent role of cytochrome P450 monooxygenase-catalyzed reactions is established.

## Introduction

Environmental plastic pollution poses a global threat to ecosystems and human health. Beyond the plastic polymers themselves, environmental and human health risks are related to the release of various additives incorporated to improve certain properties of the plastic (Hermabessiere et al., 2017; Hahladakis et al., 2018). Phthalate esters (PEs, phthalates) represent a prominent group of persistent organic micropollutants. Structurally, PEs are dialkyl or alkyl aryl esters of benzene-1,2-dicarboxylic (phthalic) acid, differing by their side chain moiety length which commands their hydrophobicity (Gao and Wen, 2016; Ren et al., 2018). The worldwide and environmental ubiquity of these compounds arises from their extensive use as plastic additives (plasticizers) to provide flexibility in the manufacturing of plastic products such as polyvinyl chloride, and as a common additive in various consumer products (i.e. cosmetics, paints, lubricants, adhesives, insecticides, packaging) (Gao and Wen, 2016). Loss can occur at any stage of product lifestyle, with chemical leaching particularly relevant to plasticizers as the absence of covalent binding to the plastic resin leaves their migration unhindered (Cartwright et al., 2000; Gao and Wen, 2016; Hahladakis et al., 2018). Consequently, PE levels are highest in urbanized areas and those directly within the vicinity of production (Cartwright et al., 2000; Liang et al., 2008; Sun et al., 2013). Careless disposal coupled with atmospheric deposition and rainfall transfer has led to widespread environmental contamination. PEs have been recorded in atmospheric samples from remote regions of the Atlantic and Arctic Oceans, and are also found in human breast milk, blood, and urine (Net et al., 2015). A study published in the International Journal of Hygiene and Environmental Health detected such compounds in urine samples of a remote Bolivian forager-horticulturist group, suggesting widespread exposure even out with industrialized populations (Sobolewski et al., 2017).

Structural parallels between PEs and the hormone estrogen have led to investigations into their xenoestrogenicity and potential activity as endocrine disrupting chemicals (EDCs). EDCs interfere with the normal homeostatic balance of a spectrum of biological processes, particularly those linked with development and reproduction (Diamanti-Kandarakis et al., 2009). Significant crossover of several PEs and the natural estrogen 17β-estradiol (E2) was profiled in regards to downstream gene expression using high-throughput screening via DNA microarray analysis (Parveen et al., 2008). A number of *in vivo* and epidemiological studies have highlighted trends between PE exposure in human populations and negative manifestations witnessed in male and female reproductive development (Colon et al., 2000; Diamanti-Kandarakis et al., 2009; Jeng, 2014). Similar adverse effects have been reported in terrestrial and aquatic wildlife populations, including annelids and molluscs which are both phyla of ecological importance (Oehlmann et al., 2009).

Bacteria are capable of degrading PEs under aerobic and anaerobic conditions, with many utilizing them as sole sources of carbon and energy for growth. Partial degradation yielding breakdown products such as phthalic acid (PA) or benzoate (BA), and the failure to grow on such PE-derived metabolites has also been reported for certain bacterial species (Liang et al., 2008; Gao and Wen, 2016; Ren et al., 2018). Comparatively, fungal PE degradation has received less attention. This statement is particularly applicable to filamentous fungi dwelling within aquatic environments and also yeasts, with previous fungal based studies predominantly focusing on terrestrial ligninolytic and non-ligninolytic species (Liang et al., 2008; Luo et al., 2012; Gao and Wen, 2016; Pezzella et al., 2017). A few studies describe fungal growth on di-2-ethylhexyl phthalate (DEHP) and dimethyl phthalate esters (DMPEs) when present as the sole carbon and energy source (Luo et al., 2011; Pradeep and Benjamin, 2012). Nevertheless, the majority of the corresponding reports point to a predominance of cometabolic PE biotransformation in fungi (Gartshore et al., 2003; Lee et al., 2007; Hwang et al., 2012; Luo et al., 2012; Ahuactzin-Perez et al., 2016; Ahuactzin-Perez et al., 2018).

Generally, microbial metabolism of PEs involves the primary biotransformation of the phthalic diester (parent compound) via a monoester form into PA, followed by complete mineralization of PA into CO_2_ and H_2_O (Staples et al., 1997; Liang et al., 2008; Ren et al., 2018). De-esterification of PEs (i.e. the enzymatic removal of alkyl chains from the PA moiety leading to the formation of carboxylic groups) can proceed via either hydrolytic cleavage or oxidative *O*-dealkylation. The successive enzymatic hydrolysis of phthalate diesters to the corresponding monoesters and then to PA is the most common PE transformation pathway in bacteria under both aerobic and anaerobic conditions, having also been reported for fungi (Lee et al., 2007; Liang et al., 2008; Ahuactzin-Perez et al., 2016, 2018; Ren et al., 2018). The alternative breakdown pathway involving alkyl chain removal via O-dealkylation is known to be catalyzed by cytochrome P450 monooxygenase systems in mammals, however it is not well understood in fungi and prokaryotes (Lee et al., 2007; Liang et al., 2008; Girvan and Munro, 2016; Cantú Reinhard and De Visser, 2017). Additionally, phthalates with longer side chains than diethyl phthalate (DEP) may first undergo alkyl chain shortening via β-oxidation, a process initiated by cytochrome P450 mediated hydroxylation, prior to de-esterification occurring (Amir et al., 2005; Liang et al., 2008). Lastly, transesterification involving the introduction of shorter alkyl chains in exchange for longer ones may alternatively take place, followed by de-esterification to yield PA as a central intermediate (Jackson et al., 1996; Cartwright et al., 2000; Lee et al., 2007; Liang et al., 2008).

To our knowledge, previous research aiming at fungal PE metabolism has either solely emphasized hydrolytic de-esterification as the primary step (Kim et al., 2003, 2005; Kim and Lee, 2005; Ahn et al., 2006; Luo et al., 2011, 2012; Hwang et al., 2012; Ahuactzin-Perez et al., 2016, 2018), or did not further elucidate the mechanisms of the suggested primary de-esterification step(s) (i.e. hydrolysis or oxidative *O*-dealkylation) (Lee et al., 2007). Furthermore, extracellular fungal oxidoreductases such as laccase and lignin-modifying peroxidases are not known to act on PEs directly, and extracellular unspecific peroxygenases (UPOs) of fungi have been reported to oxidize only a few PEs albeit slowly (Hwang et al., 2012; Macellaro et al., 2014; Karich et al., 2017; Pezzella et al., 2017). Thus, hydrolytic enzymes (e.g. cutinases, esterases, lipases) and intracellular oxidative cytochrome P450s can be considered as primary candidates for initiating the biocatalytic breakdown of PEs in fungi (Kim et al., 2005; Ghaly et al., 2010; Okamoto et al., 2011; Ahuactzin-Perez et al., 2016; Cantú Reinhard and De Visser, 2017; Ren et al., 2018), with major evidence for the latter remaining to be established. Aside from enzymatic removal, biosorption via physio-chemical processes such as adsorption, absorption, and ion interactions may also contribute and must be accounted for when investigating PE fate within the environment (Liang et al., 2008; Gadd, 2009; Net et al., 2015). Fungal biosorption of PEs and other hydrophobic environmental pollutants is a well-known phenomenon (Lee et al., 2007; Hofmann and Schlosser, 2016).

In this study we aim to broaden the scope of fungal biocatalytic and biosorptive removal of PEs from the environment through a comparative assessment of marine-, freshwater-, and terrestrial-derived asco- and basidiomycetes, i.e. members of the two major fungal groups harboring most of the known fungal pollutant degraders (Harms et al., 2011). Di-*n*-butyl phthalate (DBP) and DEP were chosen as target compounds as they represent environmentally prominent PE pollutants with differing structures and hydrophobicities (Net et al., 2015; Hermabessiere et al., 2017; Hahladakis et al., 2018; Salaudeen et al., 2018). Owing to its recalcitrant nature and recognition as a priority regulated pollutant by the European Union (Directive 2011/65/EU revision 2015/863; EU, 2015) and the U.S. Environmental Protection Agency (EPA) (Gao and Wen, 2016), additional focus was placed upon investigating removal of this compound. Previously reported fungal biotransformation metabolites of DBP have been attributed to de-esterification, hydrolysis, and transesterification reactions, with little known about possible oxidative breakdown mechanisms (Kim and Lee, 2005; Lee et al., 2007; Luo et al., 2012). Nevertheless, the longer alkyl chains of DBP compared to DEP could potentially make the compound susceptible to oxidative fungal biotransformation reactions such as *ß*-oxidation (Amir et al., 2005; Liang et al., 2008). We have therefore chosen to employ DBP as a model compound to investigate the potential role of cytochrome P450 monooxygenase reactions in fungal PE biotransformation, via the application of the cytochrome P450 inhibitor piperonyl butoxide (PB) in conjunction with mass spectrometry based structural elucidation of biotransformation products. This inhibitor was chosen due to its prior use in assessing the role of cytochrome P450s in fungal biotransformation of EDCs (Subramanian and Yadav, 2009), pharmaceutical residues (Marco-Urrea et al., 2009), pesticides and other xenobiotic compounds (Syed and Yadav, 2012; Coelho-Moreira et al., 2018). Bisphenol A (BPA), another extensively used plastic additive and prominent environmental micropollutant established as an EDC (Hermabessiere et al., 2017; Hahladakis et al., 2018), was included as a reference compound due to its well documented degradation by both, extracellular (laccase, peroxidases) and intracellular (cytochrome P450 monooxygenases) fungal enzymes (Cajthaml, 2015; Hofmann and Schlosser, 2016; Im and Löffler, 2016). Owing to the reported environmental co-occurrence of BPA and PEs (Tran et al., 2015; Notardonato et al., 2019) fungal biotransformation and biosorption capacities were assessed upon employing BPA, DBP, and DEP in mixture, hereby verifying the reported fungal peculiarity to attack multiple pollutants simultaneously (Harms et al., 2011). Extracellular laccase and peroxidase activities were concomitantly monitored in these experiments due to a possible role of such enzymes in BPA oxidation. Pathways for the fungal and bacterial metabolism of BPA are well established (Wang et al., 2014; Cajthaml, 2015; Im and Löffler, 2016), and hence were not the focus of the present study.

## Materials and Methods

### Chemicals

Unless stated otherwise all chemicals used were of analytical grade, or in the case of chromatographic solvents, gradient grade or ULC/MS grade (mass spectrometry). Bisphenol A (BPA, purity 95%) was provided by Fluka (Sigma-Aldrich, St. Louis, MO, United States; now belonging to Merck Group, Darmstadt, Germany). Dibutyl phthalate (DBP, purity > 99%), diethyl phthalate (DEP, purity 99.5%), and piperonyl butoxide (PB) of technical grade (purity 90%) were purchased from Sigma-Aldrich. 2,2′-Azinobis-(3-ethylbenzothiazoline-6-sulfonic acid) (ABTS, purity > 98%) was obtained from AppliChem (Darmstadt, Germany). All other chemicals were purchased from Merck, Sigma-Aldrich and Th. Geyer GmbH (Renningen, Germany).

### Source, Identification and Maintenance of Fungal Strains

The fungal strains employed within the present study are compiled in Table 1. They were chosen as representatives of different ecophysiological groups and were derived from various, and in cases, rarely investigated habitats exhibiting diverse environmental conditions. The strains 1-DS-2013-S2 and 1-DS-2013-S4 were isolated from sand containing algal debris from the alluvial zone and algae growing on breakwater groins, respectively. Corresponding samples used for fungal isolation were acquired from a beach in the region of Wustrow (Mecklenburg-Western Pomerania, Germany) on the Baltic Sea (coordinates: 54°21′32.246′′N, 12°23′39.725′′E) in 2013. Pure fungal cultures were obtained using a previously described procedure (Junghanns et al., 2008a), and sent to the Belgian Coordinated Collections of Microorganisms/Mycothèque de L’Université catholique de Louvain (BCCM/MUCL, Louvain-la-Neuve, Belgium) for identification. According to DNA sequencing and morphological examination, strain 1-DS-2013-S2 is a member of the Helotiales (Leotiomycetes) and considered to be of the genus *Ascocoryne*. *Ascocoryne* sp. 1-DS-2013-S2 is related to other Helotiales members originating from aquatic habitats (freshwater and marine sediments). Strain 1-DS-2013-S4 was identified as *Paradendryphiella arenariae* of the *Pleosporaceae*, a species well known from decaying marine and estuarine plants, and beach sands (Overy et al., 2014).

**TABLE 1:** Overview of fungal strains employed in the present study.

**Fungal strain**	**Phylogeny (phylum, class, order)**	**Habitat/characteristics**	**References**
*Acephala* sp. strain JU-A-2 (DSM 27592)	Ascomycota, Leotiomycetes, Helotiales	Peatland isolate	Singh et al., 2014
*Ascocoryne* sp. strain 1-DS-2013-S2	Ascomycota, Leotiomycetes, Helotiales	Marine isolate	This study
*Clavariopsis aquatica* De Wild. strain WD(A)-00-01	Ascomycota, Sordariomycetes, Microascales	Aquatic hyphomycete, freshwater isolate	Junghanns et al., 2008a
*Paradendryphiella arenariae* (Nicot) Woudenberg and Crous strain 1-DS-2013-S4	Ascomycota, Dothideomycetes, Pleosporales	Marine isolate	This study
*Phoma* sp. strain UHH 5-1-03 (DSM 22425)	Ascomycota, Dothideomycetes, Pleosporales	Freshwater isolate	Junghanns et al., 2008a
*Stachybotrys chlorohalonata* Andersen and Trane strain A-2008-2 (DSM 27588)	Ascomycota, Sordariomycetes, Hypocreales	Constructed wetland isolate	Singh et al., 2014
*Stropharia rugosoannulata* Farlow ex Murrill DSM 11372	Basidiomycota, Agaricomycetes, Agaricales	Litter inhabiting, causes white-rot decay of lignocellulose	Singh et al., 2014
*Trichosporon porosum* (Stautz) Middelhoven, Scorzetti and Fell strain JU-K-2 (DSM 27593)	Basidomycota, Tremellomycetes, Trichosporonales	Peatland isolate	Singh et al., 2014

*Strains without a DSM accession number, which denotes deposition at the German Collection of Microorganisms and Cell Cultures (DSMZ; Braunschweig, Germany), are available from the strain collection of the Department of Environmental Microbiology at the Helmholtz Centre for Environmental Research - UFZ (Leipzig, Germany). The provided phylogenetic information was derived from the NCBI taxonomy database (https://www.ncbi.nlm.nih.gov/Taxonomy/Browser/wwwtax.cgi).*

**FIGURE 1:**
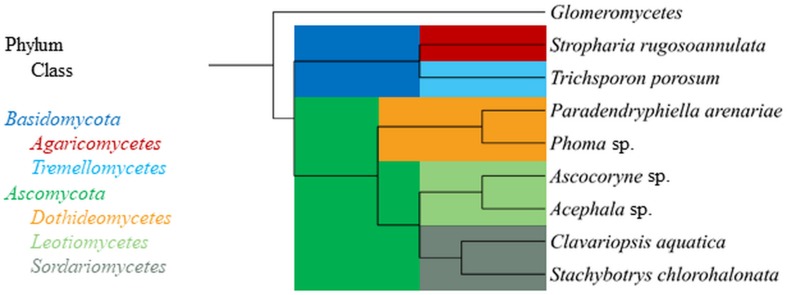
Phylogenetic tree displaying relationship of fungal strains used in this study based on NCBI taxonomy data (https://www.ncbi.nlm.nih.gov/taxonomy), generated in phyloT (https://phylot.biobyte.de/) and visualized with iTOL (http://itol.embl.de/). The class Glomeromycetes (Tedersoo et al., 2018) is shown as an outgroup.

The aquatic hyphomycete *Clavariopsis aquatica* strain WD(A)-01 represents an exclusively aquatic species frequently observed in rivers and streams (Junghanns et al., 2008a; Krauss et al., 2011). *Phoma* sp. strain UHH 5-1-03 (DSM 22425) has an ascomycete affiliation and originates from the Saale river, Germany (Junghanns et al., 2008a). *Stachybotrys chlorohalonata* strain A-2008-2 (DSM 27588) and *Acephala* sp. strain JU-A-2 (DSM 27592) were previously isolated from a constructed wetland and peatland, respectively (Singh et al., 2014), and represent fungal taxa known from several terrestrial habitats. The anamorphic yeast *Trichosporon porosum*, with the herein applied strain JU-K-2 (DSM 27593) likewise being derived from peatland (Singh et al., 2014), is also typical for terrestrial environments and related to the loubieri/laibachii group of species that assimilate hemicelluloses and phenolic compounds (Middelhoven et al., 2001). *Stropharia rugosoannulata* is a well-characterized terrestrial litter-inhabiting basidiomycete species causing white-rot decay of lignocellulosic materials (Schlosser and Hofer, 2002; Singh et al., 2014). The phylogenetic relationships among the fungal strains employed in this study are compiled in Figure 1.

Fungal strains were maintained on agar plates containing 2% malt extract (w/v) solidified with 1.5% agar (pH 5.7). For the marine-derived fungal isolates, further 2% malt extract agar plates were supplemented with an artificial seawater component, which was composed of 23.93 g/L NaCl, 10.83 g/L MgCl_2_ × 6 H_2_O, 4.01 g/L Na_2_SO_4_, 1.52 g/L CaCl_2_ × 2 H_2_O, 677 mg/L KCl, 196 mg/L NaHCO_3_, 98 mg/L KBr, 26 mg/L H_3_BO_3_, 24 mg/L SrCl_2_ × 6 H_2_O, and 3 mg/L NaF (Kester et al., 1967). These plates were employed in order to mimic conditions of the marine environment (indicated in the text where applicable). All plates were incubated at 20°C in the dark.

### Assessment of Fungal Micropollutant Removal Rates

Pre-cultures were established for subsequent batch tests with active and NaN_3_-inactivated fungal cultures in 100-mL Erlenmeyer flasks containing 30 mL of a 2% (w/v) malt extract medium (pH 5.7) (Hofmann and Schlosser, 2016). Deviating from this, 30 mL of a defined nitrogen-limited medium containing 56 mM glucose and 1.2 mM diammonium tartrate as carbon and nitrogen sources (Schlosser and Hofer, 2002; Krueger et al., 2015b) was also used for fungal pre-cultivation where indicated in the text. Further pre-cultivation media used for marine-derived fungal strains in a series of experiments (see below) additionally contained the aforementioned artificial seawater component according to (Kester et al., 1967). For certain experiments, pre-cultivation media was additionally supplemented with 50 μM CuSO_4_ and 1 mM vanillic acid to stimulate laccase production (Junghanns et al., 2008b; Hofmann and Schlosser, 2016) (indicated where applicable below). Each flask was inoculated with 0.5 mL of a mycelial suspension, which was prepared from corresponding fungal agar plate cultures (Junghanns et al., 2008b; Hofmann and Schlosser, 2016). Thereafter, the flasks were incubated on a rotary shaker (New Brunswick^TM^ Innova 44; Eppendorf, Hamburg, Germany) at 20°C and 120 rpm in the dark. After 6 days of incubation, half of the growing fungal cultures were inactivated with 1 g/L NaN_3_. Following a further day of incubation, fungal biomass was separated from pre-culture media for transfer to pollutant removal flasks by centrifugation in 50-mL conical tubes at 7197 × *g* and 20°C for 10 min (Eppendorf centrifuge 5430R, rotor FA-35-6-30, Eppendorf, Hamburg, Germany). After discarding supernatant, the biomass pellet was washed with 30 mL of a synthetic mineral salts medium devoid of a source of carbon and energy (pH 6.8; Stanier et al., 1966) and separated in a second centrifugation step. The resulting supernatant was discarded again and the biomass pellet was transferred into new 100-mL Erlenmeyer flasks containing 30 mL of the mineral salts medium mentioned before, which had been supplemented with the cytochrome P450 inhibitor PB and/or micropollutant(s) prior to fungal biomass addition.

One series of experiments addressed the contribution of fungal biotransformation and biosorption processes to overall DBP removal, and the influence of the cytochrome P450 inhibitor PB on DBP removal in a resting cell approach employing the aforementioned mineral salts medium, similarly as applied before (Hofmann and Schlosser, 2016). DBP was added to mineral medium from a stock solution prepared in methanol containing 10% (w/v) Tween 80 (included to improve compound aqueous solubility), to yield a final concentration of 62.5 μM (procedure modified from Hofmann and Schlosser, 2016). Further DBP- and mineral medium-containing flasks were additionally supplemented with PB, which was added from a stock solution in methanol with 10% Tween 80 yielding a final concentration of 1 mM. Thereafter, active fungal biomass was added as described before. Control experiments intended to assess the biosorption share of overall DBP removal were established by adding previously NaN_3_-inactivated fungal biomass (see before) to flasks with DBP-containing mineral media, which in addition had been amended with NaN_3_ at 1 g/L. Fungal biomass was omitted in further negative controls, which were amended with PB and/or DBP only. For comparability, final methanol and Tween 80 concentrations in these experiments were always adjusted to 1% (v/v) and 0.1% (w/v), respectively.

Additional experiments aiming at the verification of fungal micropollutant biotransformation and biosorption capacities under more complex conditions intended to match real environmental situations more closely were also carried out. Two types of fungal pre-culture media were applied, i.e. the defined nitrogen-limited medium and the complex 2% malt extract medium described before (Schlosser and Hofer, 2002; Krueger et al., 2015b; Hofmann and Schlosser, 2016). Both media were additionally supplemented with 50 μM CuSO_4_ and 1 mM vanillic acid to stimulate laccase production (Junghanns et al., 2008b; Hofmann and Schlosser, 2016), due to the potential involvement of this enzyme in BPA biotransformation (Cajthaml, 2015; Im and Löffler, 2016). For marine-derived strains, the artificial seawater component (Kester et al., 1967) was included in the respective media used for agar plate-based inoculum production, liquid pre-cultivation, and subsequent pollutant removal experiments (see above). The micropollutants BPA, DBP and DEP were applied in mixture and added from a combined stock solution (prepared in methanol containing 10% Tween 80 in addition). Final individual micropollutant concentrations were 62.5 μM, corresponding to final sum methanol and Tween 80 concentrations of 0.5 and 0.05%, respectively. Control experiments employing NaN_3_-inactived fungal biomass, and control experiments omitting fungal biomass were carried out as described above.

Fungal cultures were always incubated at 20°C and 120 rpm in the dark, and sampled for ultra performance liquid chromatography (UPLC) analysis of micropollutant concentrations, extracellular laccase and peroxidase activity measurements (only for experiments employing pollutant mixtures), and fungal dry biomass determination (see below) at the time points indicated in the text. Triplicate experiments were always performed.

Respective micropollutant removal rates were determined for each fungal strain. Pseudo-first-order kinetics were assumed for the removal of micropollutants from solution (Hofmann and Schlosser, 2016) following Equation 1.

(1) $$v_{\text{t}}=c_{\text{t}}*k^{\prime }$$

where the removal rate *v*_t_ (μM/h) at a given time point t in is directly proportional to the micropollutant concentration *c*_t_ (μM) at time point t, and *k’* (1/h) represents the corresponding apparent first-order decay constant. Data of pollutant concentrations versus time (means from triplicate experiments) were fitted using non-linear regression (Equation 2) without error weighting, using OriginPro software (version 2018 95G b9.5.1.195; OriginLab Corp., Northampton, MA, United States).

(2) $$c_{\text{t}}=c_{\text{a}}+c_{\text{s}}*e^{-k^{\prime }\,t}$$

In the exponential fit function (Equation 2), *c*_a_ (μM) represents a bottom asymptote micropollutant concentration approached at infinite time where micropollutant removal was incomplete, *c*_s_ corresponds to the removal rate-governing micropollutant concentration at *t* = 0 (μM) (with the sum of *c*_a_ and *c*_s_ yielding the initial micropollutant concentration), and t is the time of incubation in presence of micropollutant (h). Where micropollutant removal was achieved within the duration of the experiment, *c*_a_ was set to 0 μM. The initial (maximal) removal rates at *t* = 0 were obtained by multiplying respective *c*_s_ and *k’* values. Obtained volume-based initial removal rates were normalized using the initial fungal dry biomass concentrations (g/L) of active and inactive fungal cultures, thereby obtaining specific initial rate values (nmol/h/g). A positive difference in specific initial removal rates between active and inactive cultures was taken to indicate the specific rate of fungal biotransformation.

Initial biomass-specific pollutant removal rates represent maximal rates operative at maximal pollutant concentrations (i.e. at the time point of pollutant addition). Removal rates for a given pollutant are expected to decline as pollutant concentrations decrease, due to species specific first-order decay constants and sorption characteristics, and in the case of active fungal cultures possibly also a decrease in physiological activity resulting from nutrient depletion (Martin et al., 2007, 2009; Hofmann and Schlosser, 2016). Hence, initial pollutant removal rates derived from exponential regression of data are not linearly correlated with the amounts of pollutants removed during longer time periods. A sufficient exponential regression fit (coefficient of determination (COD) value < 0.9) could not be obtained in every situation. In addition, initial removal rates calculated from formally successful exponential regressions were sometimes artificially high due to a steep curve cut by a bottom asymptote. Data flawed by such complications was not further considered. To ensure comparability of pollutant removal rates for each fungal strain tested, and to account for the aforementioned difficulties, removal rates based on reduction of micropollutant concentration over selected time periods (based on triplicates) were manually calculated according to Equation 3.

(3) $$v_{0-t}=\left(c_{0}-c_{\text{t}}\right)/\left(t-0\right)$$

where *v*_0__–t_ represents the average removal rate over a given time period, *c*_0_ (μM) corresponds to the micropollutant concentration at *t* = 0, and *c*_t_ the micropollutant concentration at the time point of consideration as indicated in the text. Overall rates were obtained for micropollutant removal over the full experiment duration. The obtained volume-based removal rates were biomass-normalized as described for the initial (maximal) removal rates before, thereby obtaining specific rates (nmol/h/g). A positive difference in specific removal rates between active and inactive cultures was taken to indicate the specific rate of fungal biotransformation.

### Formation of DBP Biotransformation Products

In order to investigate the formation of biotransformation products from DBP and to assess the influence of the cytochrome P450 inhibitor PB on these processes, fungi were pre-cultivated on liquid 2% malt extract medium for a total of 7 days as described before (with inactivation of half of the fungal cultures by addition of 1 g/L NaN_3_ on culture day 6). Thereafter, fungal biomass was transferred to new flasks containing PB- and/or DBP-amended mineral medium as already explained above. PB and/or DBP was added from methanolic stock solutions to yield final concentrations of 1 mM and 250 μM, respectively. Tween 80 was omitted in these experiments, in order to avoid interferences in the subsequent mass spectrometry-based analyses (see below). Control experiments employing NaN_3_-inactived fungal biomass were carried out as above. Fungal cultures were incubated at 20°C and 120 rpm in the dark as described before and sampled for analysis of biotransformation metabolites at the time points indicated in the text. Triplicate experiments were always performed. Ultra performance liquid chromatography-quadrupole time-of-flight mass spectrometry (UPLC-QTOF-MS) analysis of DBP biotransformation products was performed as described in the Supplementary Material.

### Analysis of Micropollutant Concentrations Using Ultra Performance Liquid Chromatography (UPLC)

Aqueous samples (0.5 mL) taken from cell-free supernatants of fungal cultures at the time points indicated in the text were placed in 1.5-mL Eppendorf tubes, supplemented with 0.5 mL methanol, thoroughly mixed and stored at −20°C until further use (Hofmann and Schlosser, 2016). Prior to UPLC analysis, samples were thawed and subjected to centrifugation at 20817 × g and 4°C for 10 min (Eppendorf centrifuge 5430R, rotor type FA-45-30-11; Eppendorf, Hamburg, Germany) to ensure biomass free supernatant. UPLC analysis was carried out using an Aquity^TM^ UPLC system (Waters, Eschborn, Germany) equipped with an Aquity^TM^ UPLC BEH C18 column (1.7 μM particle size, 2.1 × 50 mm; Waters) as described before (Hofmann and Schlosser, 2016), with the following modifications. Eluent A consisted of 10% (v/v) methanol in deionized water (Q-Gard 2, Millipore, Schwalbach, Germany) and eluent B of methanol, both acidified to pH 3.0 with 0.1% (v/v) formic acid. The following elution profile was applied for DBP analysis in experiments employing DBP as a single pollutant: isocratic elution at 30% B for 0.14 min, linear increase to 35% B until 5 min, further linear increase to 99.9% B until 5.5 min, isocratic elution at 99.9% B until 8.0 min, linear decrease to 30% B until 8.2 min, and isocratic elution at 30% B until 8.5 min (0.5 mL/min flow rate). For analysis of pollutant mixtures consisting of BPA, DBP and DEP, the elution profile was modified as follows: isocratic elution at 30% B for 0.14 min, linear increase to 30% B until 5 min, further linear increase to 35% B until 5.5 min, again linear increase to 99.9% B until 7.0 min, isocratic elution at 99.9% B until 7.2 min, linear decrease to 30% B until 7.5 min (0.5 mL/min flow rate). The detection wavelength was set to 278 nm. The methods were calibrated using external standards.

### Determination of Extracellular Laccase and Peroxidase Activities in Supernatants of Fungal Cultures

Laccase activity was routinely determined following the oxidation of 2 mM ABTS in McIlvaine buffer (pH 4.0) at 420 nm, as previously described (Junghanns et al., 2008b; Hofmann and Schlosser, 2016). Peroxidase activity was determined following the oxidation of 2 mM ABTS in presence of 100 μM H_2_O_2_ and 1 mM ethylenediaminetetraacetate (EDTA) disodium salt in 50 mM sodium malonate buffer (pH 4.5) (peroxidase procedure modified from Schlosser et al., 2000). Peroxidase activities were corrected for laccase activities through omitting H_2_O_2_. Enzyme activities are expressed in international units (U), where 1 U is defined as the amount of enzyme capable of oxidizing 1 μmol ABTS per minute. All enzymatic assays were performed using a GENios Plus microplate reader (Tecan, Männedorf, Switzerland).

### Fungal Dry Biomass Determination

Fungal dry biomasses of the active and NaN_3_-inactivated fungal cultures were determined at the time point of micropollutant addition, using a gravimetric procedure previously described (Hofmann and Schlosser, 2016).

## Results

### Micropollutant Removal by Fungal Cultures

#### Fungal DBP Removal and Influence of PB

**FIGURE 2:**
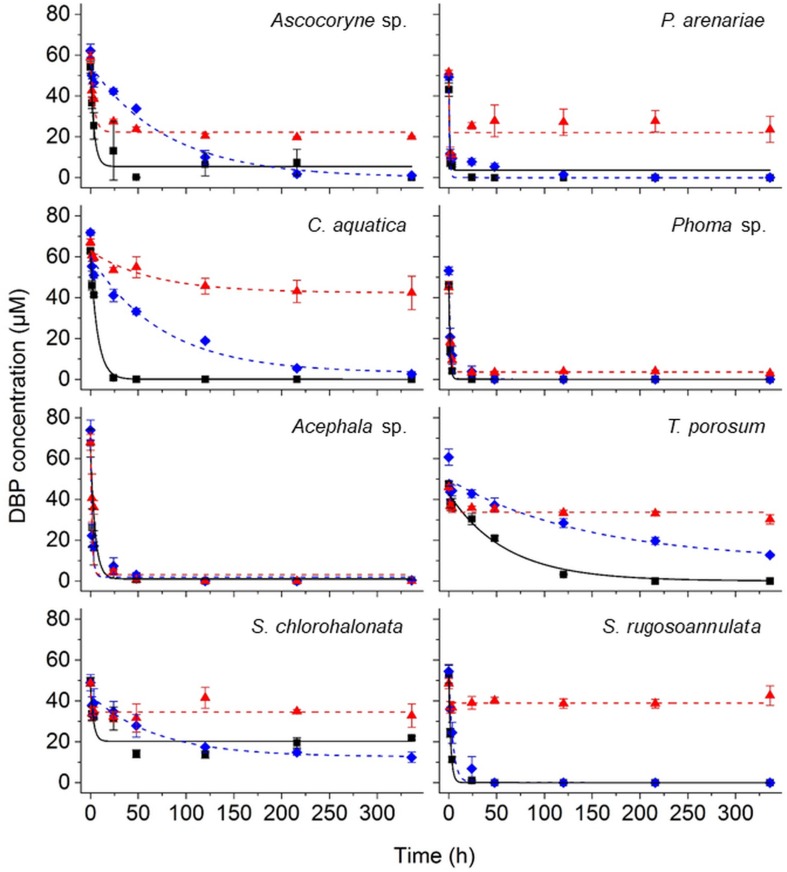
Time courses of DBP concentrations in active (black squares), PB-inhibited (blue diamonds), and NaN_3_-inactivated cultures (red triangles) of the marine-derived strains *Ascocoryne* sp. and *P. arenariae*, the freshwater-derived strains *C. aquatica* and *Phoma* sp., the peatland isolates *Acephala* sp. and *T. porosum*, the constructed wetland isolate *S. chlorohalonata*, and the litter-inhabiting basidiomycete *S. rugosoannulata*. The corresponding lines (solid black, dotted blue, and dotted red lines for active, PB-inhibited, and NaN_3_-inactivated cultures, respectively) arise from data fitting of measured DBP concentrations as described in the materials and methods section. Symbols represent means ± standard deviations from triplicate cultures.

The removal of DBP was followed by UPLC analysis of fungal culture supernatants. DBP was removed to varying degrees depending on the respective fungal strain and cultivation condition (Figure 2 and Table 2). In active fungal cultures (i.e. those omitting PB and NaN_3_), the DBP concentration had decreased to approximately 31 μM and values below the quantification limit within 3.5 h of incubation, corresponding to relative removals (i.e. in relation to the actual quantified initial concentration) of approximately 36–100% (Figure 2). With the exception of *S. chlorohalonata* (approximately 44% of the initial concentration remaining), DBP was completely removed by active fungal cultures within 14 days (336 h; Figure 2). In PB-inhibited cultures, DBP concentrations were reduced to values ranging from 51 to 9 μM after 3.5 h, corresponding to a relative reduction of approximately 21–81% already within this time period. Similarly within 3.5 h of incubation, 22–94% of DBP was absent from the sampled supernatant of NaN_3_-inactivated fungal cultures, suggesting a considerable influence of biosorption on the reduction of DBP concentration. In fungal biomass-free negative controls, the initially applied DBP concentration remained constant over the whole duration of the experiment (data not shown).

**TABLE 2:** DBP removal rates and influence of PB on DPB removal observed with fungal cultures.

**Fungus and mode of rate calculation**	**Removal rate (nmol/h/g)**				**PB inhibition (%)**
	**Active**	**NaN_3_-inactivated**	**Biotransformation**	**PB-inhibited**	
***Ascocoryne* sp.**					
Initial rate	5442 ± 1773	7649 ± 2266	0	295 ± 62	95 ± 37
24 h rate	738 ± 224	1071 ± 132	0	359 ± 52	51 ± 17
Overall rate	70 ± 9	95 ± 17	0	79 ± 9	0
***P. arenariae***					
Initial rate	5095 ± 852	FNS	NA	5486 ± 1670	0
24 h rate	205 ± 21	179 ± 15	26 ± 26	198 ± 17	3 ± 0
Overall rate	15 ± 2	14 ± 3	1 ± 3	17 ± 1	0
***C. aquatica***					
Initial rate	3050 ± 445	171 ± 76	2879 ± 451	280 ± 106	91 ± 37
24 h rate	954 ± 48	317 ± 20	637 ± 52	472 ± 42	51 ± 5
Overall rate	69 ± 4	41 ± 11	28 ± 12	76 ± 4	0
***Phoma* sp.**					
Initial rate	3090 ± 354	2389 ± 239	701 ± 427	2470 ± 406	20 ± 4
24 h rate	170 ± 19	156 ± 20	14 ± 28	183 ± 21	0
Overall rate	12 ± 1	11 ± 1	1 ± 2	14 ± 2	0
***Acephala* sp.**					
Initial rate	4091 ± 678	1790 ± 387	2301 ± 781	5708 ± 1273	0
24 h Rate	290 ± 12	356 ± 18	0	314 ± 10	0
Overall rate	23 ± 2	27 ± 2	0	25 ± 2	0
***T. porosum***					
Initial rate	86 ± 14	FNS	NA	FNS	NA
24 h rate	86 ± 10	62 ± 6	24 ± 11	90 ± 11	0
Overall rate	17 ± 1	7 ± 1	10 ± 1	17 ± 1	0
***S. chlorohalonata***					
Initial rate	FNS	FNS	NA	61 ± 23	NA
24 h rate	105 ± 23	131 ± 17	0	81 ± 9	22 ± 5
Overall rate	11 ± 1	9 ± 3	2 ± 3	15 ± 1	0
***S. rugosoannulata***					
Initial rate	7341 ± 585	FNS	NA	3624 ± 616	51 ± 10
24 h rate	626 ± 67	199 ± 18	427 ± 70	572 ± 94	9 ± 2
Overall rate	46 ± 5	9 ± 3	37 ± 6	47 ± 4	0

*Fungal strains were first sorted according to their origin (marine-derived, freshwater, peatland, constructed wetland, soil litter) and then in alphabetical order, in line with Figure 2. Removal rates of DBP observed with active, PB-inhibited and NaN_3_-inactivated fungal cultures (controls) were always based on corresponding fungal dry biomasses at the time point of DPB addition. The fungal dry biomasses were 2.31 ± 0.26 and 1.22 ± 0.05 (*Ascocoryne* sp.), 8.73 ± 0.68 and 6.09 ± 0.15 (*P. arenariae*), 2.71 ± 0.13 and 1.79 ± 0.12 (*C. aquatica*), 11.24 ± 1.26 and 11.17 ± 0.88 (*Phoma* sp.), 8.81 ± 0.23 and 7.44 ± 0.14 (*Acephala* sp.), 8.32 ± 0.38 and 6.77 ± 1.94 (T. porosum), 7.22 ± 0.56 and 5.19 ± 0.94 (*S. chlorohalonata*), and 3.47 ± 0.23 and 1.97 ± 0.30 g/L (*S. rugosoannulata*) (first value always for both fully active and PB-inhibited cultures, second value always for NaN_3_-inactivated cultures; values always means ± standard deviations from triplicate cultures). Initial (maximal) removal rates were calculated from exponential fits of DBP concentrations over time. Fits yielding COD values < 0.9 were considered as insufficient and initial rates were omitted in such cases (FNS, fit not sufficient). Alternatively, removal rates were also calculated based upon the reduction of DBP concentrations over time periods of 24 (24 h rate) and 336 h (end of the incubation period; overall rate). Removal rates attributed to biotransformation were expressed as the difference between the removal rates obtained from active (representing biotransformation + biosorption) and NaN_3_-inactivated fungal cultures (representing biosorption only). PB inhibition is the relative inhibition (%) of DBP removal in PB amended compared to fully active fungal cultures. Data represent means ± standard deviations (standard errors in case of initial rates and the thereof derived parameters) from triplicate cultures (calculated according to Gaussian error propagation rules). NA, not applicable.*

A comparison of fungal biomass-normalized DBP removal rates obtained from active, PB-inhibited, and NaN_3_-inactivated cultures of the investigated fungal strains revealed strain-specific contributions of biotransformation and biosorption to total DBP removal (i.e. the sum of biosorption and biotransformation) (Table 2). DBP removal attributed to biosorption was most prominent in the marine isolate *Ascocoryne* sp., and least pronounced in the yeast *T. porosum* (Table 2). After 48 h of incubation, DBP was completely removed in NaN_3_-inactivated cultures of *Acephala* sp. (Figure 2). For all other NaN_3_-inactivated fungal cultures, the DBP concentrations tended to level off over the duration of the experiment, suggesting that sorption equilibria were reached. DBP biotransformation can be deduced from the observed biomass-normalized initial DBP removal rates of fungal strains according to the following rank order: *C. aquatica* > *Acephala* sp. > *Phoma* sp. > *S. rugosoannulata* > *T. porosum*. Though suggested by the respective time courses of DBP concentrations in active and NaN_3_-inactivated fungal cultures as shown in Figure 2, biotransformation remained ambiguous upon considering the corresponding biomass-normalized DBP removal rates for *Ascocoryne* sp., *P. arenariae*, and *S. chlorohalonata*; also due to considerably high errors associated with the respective data (Table 2).

Compared to active fungal cultures, biomass-normalized DBP removal rates were reduced in the presence of the cytochrome P450 inhibitor PB by more than 50% within the first 24 h of incubation in *Ascocoryne* sp., *C. aquatica*, and *S. rugosoannulata* (Table 2). A similarly strong inhibition of DBP removal caused by PB was evident for *S. chlorohalonata* (Figure 2), even though initial DBP removal rates could not reliably be determined for active cultures of this fungus (Table 2). A comparatively weaker effect of PB on DBP removal could be inferred for *Phoma* sp. (Figure 2 and Table 2). These results clearly indicate the involvement of cytochrome P450 monooxygenases in the first biocatalytic step of DBP removal in these fungi. The influence of PB on DBP removal remained inconclusive for *Acephala* sp., *P. arenariae*, and *T. porosum*, where corresponding results may partly have been biased by the quite rapid initial DBP removal especially observed with *Acephala* sp. and *P. arenariae* (Figure 2 and Table 2).

#### Fungal Removal of Micropollutants Applied in Mixture

**FIGURE 3:**
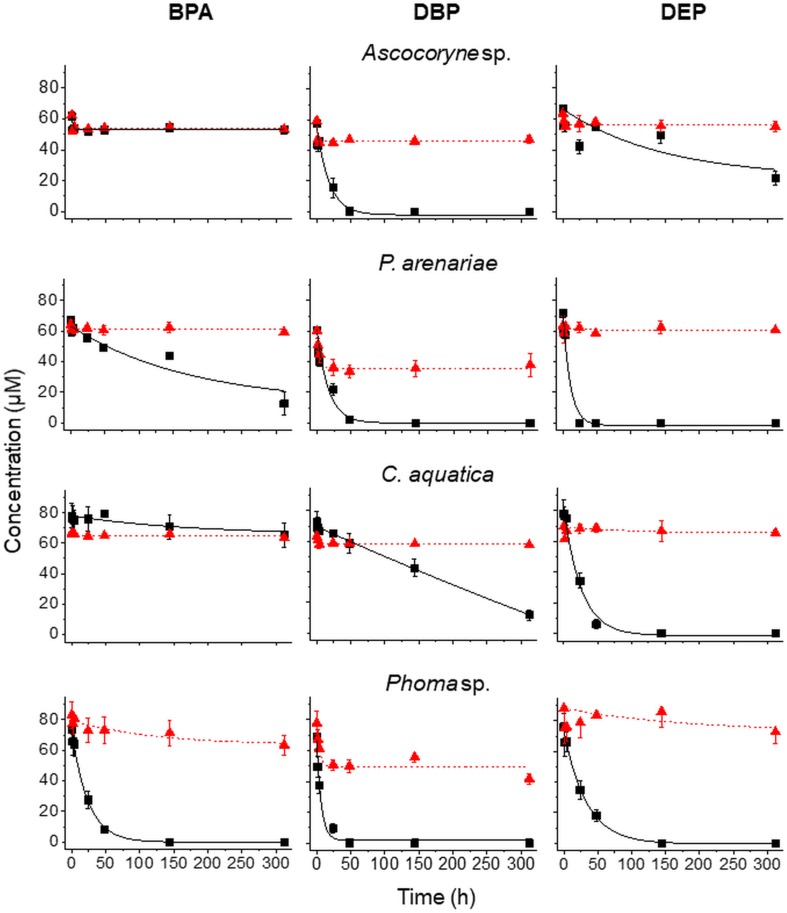
Time courses of BPA **(left)**, DBP **(middle)**, and DEP concentrations **(right)** upon compound application in mixture to active (black squares) and NaN_3_-inactivated cultures (red triangles) of *Ascocoryne* sp., *P. arenariae*, *C. aquatica*, and *Phoma* sp. (from top to bottom), which were pre-grown on defined nitrogen-limited media as described in the materials and methods section. The corresponding lines (solid black and dotted red lines for active and NaN_3_-inactivated cultures, respectively) arise from data fitting of measured compound concentrations as described in the materials and methods section. Symbols represent means ± standard deviations from triplicate cultures.

In order to verify the micropollutant biotransformation and biosorption capacities of the marine- and freshwater-derived fungal strains under more complex conditions intended to match real environmental situations more closely, pollutant mixtures composed of BPA, DBP, and DEP were applied to the fungal cultures (Figure 3 and Tables 3–6). An artificial seawater component was additionally included in media used for the marine-derived fungi (Kester et al., 1967).

**TABLE 3:** Removal rates of BPA, DBP, and DEP applied in mixture to *Ascocoryne* sp. cultures.

**Pollutant/description**	**Removal rate (nmol/h/g)**		
	**Initial rate**	**24 h Rate**	**Overall rate**
**BPA**			
Active cultures	FNR	679 ± 228	47 ± 12
NaN_3_-inactivated cultures	FNR	917 ± 252	71 ± 21
Biotransformation	NA	0	0
**DBP**			
Active cultures	4367 ± 1101	2828 ± 478	299 ± 16
NaN_3_-inactivated cultures	FNR	1452 ± 202	94 ± 25
Biotransformation	NA	1376 ± 519	205 ± 30
**DEP**			
Active cultures	FNS	1638 ± 358	232 ± 28
NaN_3_-inactivated cultures	FNS	690 ± 512	67 ± 28
Biotransformation	NA	948 ± 629	165 ± 40

*Removal rates of BPA, DBP, and DEP observed with active and NaN_3_-inactivated *Ascocoryne* sp. cultures (controls) were always based on corresponding fungal dry biomasses at the time point of addition of pollutant mixtures. The fungal dry biomasses were 0.69 ± 0.02 and 0.46 ± 0.05 g/L for active and NaN_3_-inactivated cultures, respectively (values always means ± standard deviations from triplicate cultures). Initial (maximal) removal rates were calculated from exponential fits of micropollutant concentrations over time. Fits yielding COD values < 0.9 were considered as insufficient and initial rates were omitted in such cases (FNS, fit not sufficient). Also, formally successful fits yielding unrealistic removal rates due to initial sharp decreases in micropollutant concentrations and resulting steep curve cuts by the related bottom asymptotes were not considered (FNR, fit not realistic). Alternatively, removal rates were also calculated based upon the reduction of micropollutant concentrations over time periods of 24 (24 h rate) and 312 h (end of the incubation period; overall rate). Removal rates attributed to biotransformation were expressed as the difference between removal rates obtained from active (representing biotransformation + biosorption) and NaN_3_-inactivated fungal cultures (representing biosorption only). Data represent means ± standard deviations (standard errors in case of initial rates and the thereof derived share of biotransformation) from triplicate cultures (calculated according to Gaussian error propagation rules). NA, not applicable.*

**TABLE 4:** Removal rates of BPA, DBP, and DEP applied in mixture to *P. arenariae* cultures.

**Pollutant, cultivation variant and share of biotransformation**	**Removal rate (nmol/h/g)**		
	**Initial rate**	**24 h Rate**	**Overall rate**
**BPA**			
Active cultures	FNS	168 ± 48	62 ± 10
NaN_3_-inactivated cultures	FNS	37 ± 52	6 ± 4
Biotransformation	NA	131 ± 71	56 ± 11
**DBP**			
Active cultures	1145 ± 260	572 ± 64	69 ± 4
NaN_3_-inactivated cultures	2279 ± 474	401 ± 98	29 ± 10
Biotransformation	0	171 ± 117	40 ± 11
**DEP**			
Active cultures	2198 ± 650	1059 ± 70	82 ± 5
NaN_3_-inactivated cultures	FNS	21 ± 121	4 ± 9
Biotransformation	NA	1038 ± 140	78 ± 10

*Removal rates of BPA, DBP, and DEP observed with active and NaN_3_-inactivated *P. arenariae* cultures (controls) were always based on corresponding fungal dry biomasses at the time point of addition of pollutant mixtures. The fungal dry biomasses were 3.13 ± 0.17 and 2.78 ± 0.13 g/L for active and NaN_3_-inactivated cultures, respectively (values always means ± standard deviations from triplicate cultures). For further explanations please refer to Table 3.*

**TABLE 5:** Removal rates of BPA, DBP, and DEP applied in mixture to *C. aquatica* cultures.

**Pollutant, cultivation variant and share of biotransformation**	**Removal rate (nmol/h/g)**		
	**Initial rate**	**24 h Rate**	**Overall rate**
**BPA**			
Active cultures	FNS	114 ± 579	49 ± 45
NaN_3_-inactivated cultures	FNS	213 ± 214	22 ± 14
Biotransformation	NA	0	27 ± 47
**DBP**			
Active cultures	223 ± 569	377 ± 359	241 ± 36
NaN_3_-inactivated cultures	FNS	441 ± 123	43 ± 13
Biotransformation	NA	0	198 ± 39
**DEP**			
Active cultures	3610 ± 640	2274 ± 549	311 ± 43
NaN_3_-inactivated cultures	FNS	146 ± 372	32 ± 20
Biotransformation	NA	2128 ± 663	279 ± 47

*Removal rates of BPA, DBP, and DEP observed with active and NaN_3_-inactivated *C. aquatica* cultures (controls) were always based on corresponding fungal dry biomasses at the time point of addition of pollutant mixtures. The fungal dry biomasses were 0.90 ± 0.07 and 0.47 ± 0.05 g/L for active and NaN_3_-inactivated cultures, respectively (values always means ± standard deviations from triplicate cultures). For further explanations please refer to Table 3.*

**TABLE 6:** Removal rates of BPA, DBP, and DEP applied in mixture to *Phoma* sp. cultures.

**Pollutant, cultivation variant and share of biotransformation**	**Removal rate (nmol/h/g)**		
	**Initial rate**	**24 h Rate**	**Overall rate**
**BPA**			
Active cultures	1448 ± 89	1019 ± 226	125 ± 15
NaN_3_-inactivated cultures	31 ± 40	342	44 ± 24
Biotransformation	1147 ± 97	722 ± 410	81 ± 29
**DBP**			
Active cultures	4547 ± 1032	1310 ± 148	117 ± 13
NaN_3_-inactivated cultures	FNS	799 ± 257	82 ± 20
Biotransformation	NA	511 ± 315	35 ± 24
**DEP**			
Active cultures	1053 ± 116	915 ± 231	129 ± 15
NaN_3_-inactivated cultures	FNS	276 ± 418	35 ± 29
Biotransformation	NA	639 ± 478	94 ± 33

*Removal rates of BPA, DBP, and DEP observed with active and NaN_3_-inactivated *Phoma* sp. cultures (controls) were always based on corresponding fungal dry biomasses at the time point of addition of pollutant mixtures. The fungal dry biomasses were 2.10 ± 0.03 and 1.58 ± 0.12 g/L for active and NaN_3_-inactivated cultures, respectively (values always means ± standard deviations from triplicate cultures). For further explanations please refer to Table 3.*

After fungal pre-cultivation on a previously described defined nitrogen-limited medium (Schlosser and Hofer, 2002; Krueger et al., 2015b), concomitant biotransformation of DBP and DEP by the Baltic Sea isolate *Ascocoryne* sp. was clearly evident whereas biotransformation of BPA was not apparent (Figure 3 and Table 3). Similarly, the obligate freshwater inhabitant (aquatic hyphomycete) *C. aquatica* removed substantial amounts of DBP and DEP, but not of BPA when present in mixture (Figure 3 and Table 5). Biotransformation of all applied micropollutants was indicated for the marine isolate *P. arenariae*, as well as for the freshwater-derived ascomycete *Phoma* sp. (Figure 3 and Tables 4, 6). The efficiency of biotransformation (in terms of biomass-normalized removal rates) followed the rank order *Phoma* sp. > *P. arenariae* for BPA, *Ascocoryne* sp. > *Phoma* sp. > *C. aquatica* > *P. arenariae* for DBP, and *C. aquatica* > *P. arenariae* > *Ascocoryne* sp. > *Phoma* sp. for DEP (Tables 3–6). The biotransformation rate of DBP was higher than that of DEP in *Ascocoryne* sp. (Figure 3 and Table 3), whereas the opposite was observed for the other tested fungi (Figure 3 and Tables 4–6). Biomass-normalized removal rates accounting for biosorption were generally highest for DBP (the most hydrophobic compound among the tested micropollutants), followed by those observed for BPA and DEP (Tables 3–6). For all three micropollutants the determined biosorption rates were highest in *Ascocoryne* sp., followed by *Phoma* sp., *C. aquatica*, and *P. arenariae* (Tables 3–6); similar to the results obtained from application of DBP only (Table 2).

Extracellular laccase and peroxidase activities were monitored since these enzymes are well known to oxidize BPA efficiently (Cajthaml, 2015). Laccase activities steadily increased in *Phoma* sp., with a maximum of 98.7 ± 13.2 U/L (mean ± standard deviation for triplicate cultures) after 144 h of incubation and subsequently declined to 80.5 ± 11.3 U/L at the end of the experiment (312 h of incubation). No meaningful laccase activities were detected for any other fungal strain and for NaN_3_-inactivated cultures of *Phoma* sp. (all activities measured below 1 U/L). Likewise, peroxidase activities were only detected for active *Phoma* sp. cultures. A maximal peroxidase activity was quickly established, with 38.8 ± 19.7 U/L already measurable following 48 h of incubation. This slowly declined to 34.2 ± 26.4 U/L at around 144 h of incubation, followed by a steeper decline to ∼6 U/L at the end of incubation. No evidence for peroxidase activity was obtained from the other fungal strains and for NaN_3_-inactivated cultures of *Phoma* sp.

Fungal pre-cultivation on 2% malt extract medium (Hofmann and Schlosser, 2016) in place of the defined nitrogen-limited medium reported above yielded considerably higher fungal dry biomasses for all of the tested fungi except *C. aquatica* (data not shown). Nevertheless, the biomass-normalized micropollutant removal rates and laccase activities obtained were qualitatively comparable to those observed upon fungal pre-cultivation on defined nitrogen-limited medium (data not shown), hereby confirming that the applied cultivation media had no decisive influence on the respective fundamental capacities of the tested fungi for pollutant biotransformation and biosorption. Peroxidase activities were not recorded in any of the tested fungi following this alternate pre-cultivation.

### Formation of DBP Biotransformation Products in Fungal Cultures

UPLC-QTOF-MS was applied to analyze DBP biotransformation products in fungal cultures substantiating biochemical DBP alteration as a cause for its removal, whilst concomitantly investigating effects of the cytochrome P450 inhibitor PB on metabolite formation, and allowing partial pathways for DBP metabolization by ecophysiologically diverse fungi to be established. The Baltic Sea-derived *Ascocoryne* sp., the freshwater isolate *Phoma* sp., the environmentally ubiquitous mold *S. chlorohalonata* (all ascomycetes), the terrestrial litter-decaying basidiomycete *S. rugosoannulata*, and the soil-dwelling basidiomyceteous yeast *T. porosum* were chosen for this purpose.

Tentative structures and mass spectral characteristics of DBP metabolites detected in fungal cultures with UPLC-QTOF-MS (operated in positive centroid mode) which were absent from the corresponding control cultures are listed in Supplementary Table S1. The accompanying number of the transformation products (TPs), as shown in Supplementary Table S1, indicates the respective mass of the corresponding molecular ion (always down rounded). No further DBP biotransformation products were detected using UPLC-QTOF-MS in negative electrospray ionization mode (data not shown). Low concentrations of the biotransformation metabolites did not allow for their isolation from the biological matrix in amounts sufficient for confirmation by nuclear magnetic resonance spectroscopy. Overall, the detected DBP biotransformation products comprise; (i) a range of compounds lacking additional oxygen atoms (compared to parent DBP) such as monobutyl phthalate (MBP), PA, and TPs 217 and 203; (ii) several compounds indicating the introduction of one oxygen atom (TPs 317, 315, 275, 261, 259, and 247); and (iii) a group of products containing two additional oxygen atoms in their structure (TPs 333, 331, 305, and 291; Supplementary Table S1).

**FIGURE 4:**
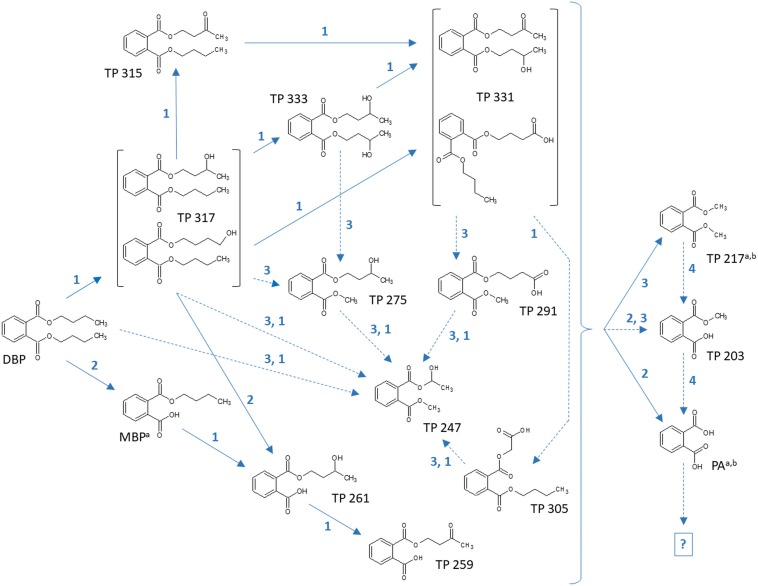
Proposed biochemical reactions summarizing the metabolization of DBP by *Ascocoryne* sp., *Phoma* sp., *S. chlorohalonata*, *S. rugosoannulata*, and *T. porosum*. Labeling of chemical metabolite structures refers to that applied in Tables 7, 8, and in Supplementary Table S1. Reaction steps indicated by solid blue arrows were deduced from respective metabolite structure proposals, relative intensities of metabolite peak areas, time courses of metabolite formation, and corresponding PB effects. Dotted blue arrows indicate possible albeit so far comparatively more speculative reaction steps. The direct precursors of TP 217, TP 203, and PA remain uncertain, due to various possibilities for the formation of these compounds (indicated by a curly bracket intended to summarize potential precursor metabolites). Biochemical alterations indicated by blue numbers comprise the cytochrome P450 monooxygenase-dependent introduction of oxygen atoms and subsequent further oxidation steps **(1)**, de-esterification via hydrolytic cleavage or oxidative *O*-dealkylation **(2)**, transesterification **(3)**, and demethylation steps **(4)** to yield PA. The further fate of PA remains uncertain (indicated by a question mark). Superscript letters associated with labels of some metabolites indicate no substantial effect of PB on the formation of the respective metabolite in *Ascocoryne* sp. **(^a^)** and *Phoma* sp. **(^b^)**.

Table 7 depicts the appearance of (i) DBP biotransformation products without additional oxygen atoms in active and PB-inhibited fungal cultures, as observed at different time points of fungal cultivation. The compounds MBP, PA, and the methylated PA derivatives, TP 217 and 203, were detected in all investigated fungal cultures. The concentrations of these DBP metabolites could not be determined since reference standards for compound quantification were not available. Therefore, peak areas of DBP biotransformation products in UPLC-QTOF-MS total ion current chromatograms were set in relation to the parent DBP peak area recorded at the time point of DBP addition (start of the experiment), respectively, and expressed as relative intensities (%). However, due to the potentially varying UPLC-QTOF-MS signal intensities of the different compounds the values thus obtained do not reflect real compound concentrations. Nevertheless, they enable direct comparisons of the respective DBP metabolite amounts within one fungus and in between different fungal species. Furthermore, these relative intensities may reasonably be expected to provide an impression about the order of magnitude of the respective quantities of the various DBP biotransformation products. The formation of MBP was clearly inhibited in the presence of PB in all fungal strains except *Ascocoryne* sp. (Table 7), suggesting a cytochrome P450 monooxygenase-dependent oxidative O-dealkylation as the major cause of the primary removal of one butyl chain from DBP in *Phoma* sp., *S. chlorohalonata*, *S. rugosoannulata*, and *T. porosum*. By contrast, the formation of MBP in *Ascocoryne* sp. in the presence of PB points to a prominent hydrolytic de-esterification, albeit without ruling out some possibly accompanying oxidative dealkylation of DBP to MBP. Additionally, the formation of PA and its potential precursor TP 217 was not inhibited by PB in *Ascocoryne* sp. and *Phoma* sp. (not shown for TP 217 formation by *Ascocoryne* sp. in Table 7, due to a generally low amount of TP 217 in this fungus). In contrast, for the other metabolites and all other tested fungi (regardless of the respective DBP metabolite produced) such an inhibition was always observed (Table 7). The direct precursors of the DBP breakdown products TP 217, TP 203, and PA could not be confirmed based on the obtained data, due to numerous possibilities for the respective reactions leading to their formation (Figure 4). Nevertheless, a positive influence of cytochrome P450-dependent reaction steps to yield the central DBP intermediate PA and its potential precursors TP 217 and 203 can be inferred for *S. chlorohalonata*, *S. rugosoannulata*, and *T. porosum*, whereas in *Ascocoryne* sp. and *Phoma* sp. transesterification and hydrolytic de-esterification reactions control the formation of TP 217 and PA, respectively.

**TABLE 7:** Fungal DBP transformation products without additional oxygen atoms in their structures.

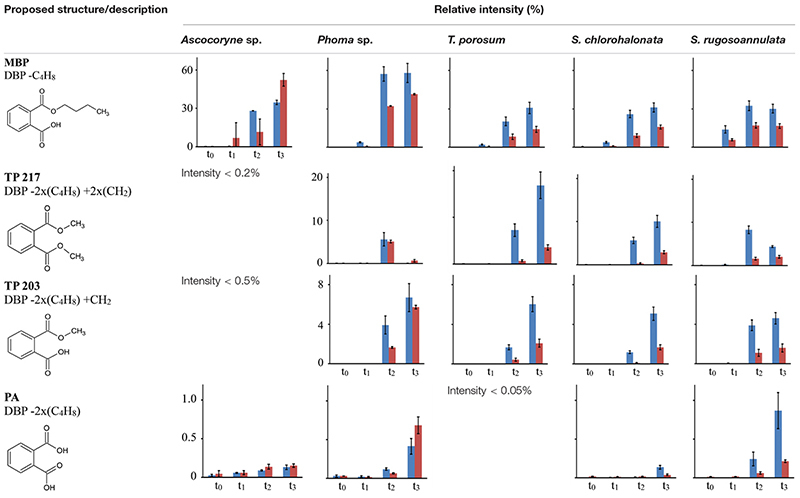

*DBP transformation products were sorted in descending order according to their molecular masses. Labeling of the proposed chemical structures and respective chemical alterations (compared to parent DBP) indicated beneath correspond to those applied in the Supplementary Table S1. Relative intensities (%) were expressed as peak areas of DBP biotransformation products in UPLC-QTOF-MS total ion current chromatograms relative to the parent DBP peak area recorded at the time point of DBP addition (start of the experiment), respectively. Blue and red bars indicate relative transformation product intensities in active and PB-inhibited fungal cultures, respectively (means ± standard deviations from triplicate cultures are always shown). For fungi displaying DBP metabolites with relative intensities below one tenth of the maximum value recorded for the metabolite of concern in any fungus, bar graphs were omitted (corresponding intensity ranges are given instead). Fungal cultures were sampled for UPLC-QTOF-MS analysis at the beginning of experiments (time point of DBP addition; t_0_), after 1.5 (*Ascocoryne* sp., *Phoma* sp.) and 3.5 h (all other fungi), respectively (t_1_), after 48 h (all tested fungi; t_2_), and at the respective termination of experiment (192 h for Ascocoryne sp. and Phoma sp.; 216 h for all other tested fungi) (t_3_).*

Time courses for the appearance of: (ii) DBP breakdown products with one additional oxygen atom (TPs 317, 315, 275, 261, 259, and 247), and (iii) two additional oxygen atoms in their structures (TPs 333 and 331; not shown for TP 305 and 291 which occurred only in small amounts) for active and PB-inhibited fungal cultures are shown in Table 8. Among these DBP biotransformation products, more than one isomer was detected for the TPs 317 (2 isomers), 275 (3), 261 (2), 333 (4), 331 (2), 305 (2), and 291 (4) (indicated by corresponding retention times in Supplementary Table S1, respectively). The occurrence of biotransformation products in the form of different isomers most likely reflects structural alterations at different carbon atom positions in the respective metabolite molecule. Related possible (albeit thus far hypothetical) structures are exemplified for TPs 331, 317, and 261 in Supplementary Table S1. Fungus-specific differences with regard to the preferential formation of certain isomers over others were noticed. However, we here abstain from presenting related data as it is impossible to assign corresponding chemical structures to the different isomers unambiguously. All of the DBP metabolites listed in Table 8 could be detected in *Ascocoryne* sp., *Phoma* sp., *S. chlorohalonata*, and *S. rugosoannulata*, whereas in *T. porosum* only the TPs 275 and 331 were found. TP formation was strongly inhibited in presence of PB as would be expected for the involvement of cytochrome P450 monooxygenase systems, which also applies to TPs 305 and 291 not shown in Table 8. Beyond primary monohydroxylation of DBP as indicated by the formation of TP 317, the deduced structures of other DBP biotransformation products depicted in Table 8 suggest further oxidation steps such as subsequent hydroxylation to dihydroxy compounds (TP 333), the formation of carbonyl or carboxyl groups (TPs 259, 315, 331), and alkyl chain shortening likely via *ß*-oxidation (TP 305). De-esterification (TP 261) and transesterification (TPs 247, 275, 291) of oxidized DBP metabolites, and further hydroxylation following transesterification (TP 247) is also supported by the proposed structures.

**TABLE 8:** Fungal DBP biotransformation products containing one or two additional oxygen atoms in their structures.

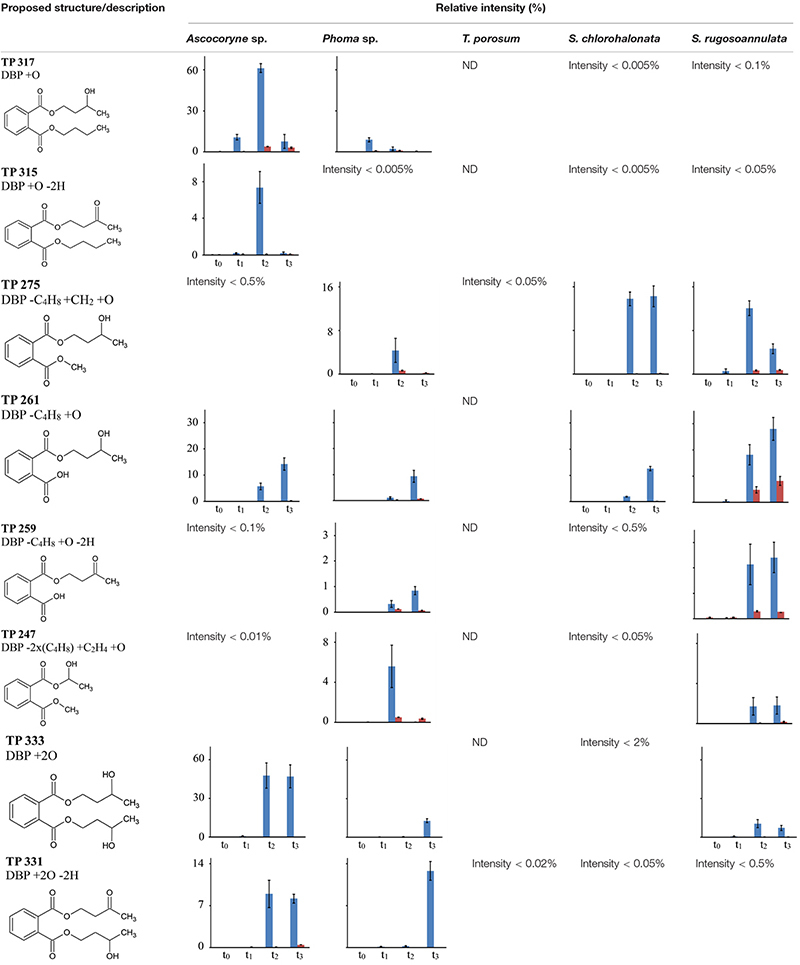

*DBP biotransformation products were grouped according to the number of additional oxygen atoms (i.e. one or two) in their structures and then sorted in descending order according to their molecular masses. Labeling of the proposed chemical structures and respective chemical alterations (compared to parent DBP) indicated beneath correspond to those applied in the Supplementary Table S1. Relative intensities (%) were expressed as peak areas of DBP biotransformation products in UPLC-QTOF-MS total ion current chromatograms relative to the parent DBP peak area recorded at the time point of DBP addition (start of the experiment), respectively. Blue and red bars indicate relative metabolite intensities in active and PB-inhibited fungal cultures, respectively (means ± standard deviations from triplicate cultures are always shown). For DBP biotransformation products occurring in the form of different isomers (see Supplementary Table S1) only the isomer appearing with the highest relative intensity, respectively, is shown. For fungi displaying DBP metabolites with relative intensities below one tenth of the maximum value recorded for the metabolite of concern in any fungus, bar graphs were omitted (corresponding intensity ranges are given instead). TPs 305 and 291 (Supplementary Table S1) are not shown because of their generally low relative intensities (below 1.0 and 0.5% in any fungus, respectively). Fungal cultures were sampled for UPLC-QTOF-MS analysis at the beginning of experiments (time point of DBP addition; t_0_), after 1.5 (*Ascocoryne* sp., *Phoma* sp.) and 3.5 h (all other fungi), respectively (t_1_), after 48 h (all tested fungi; t_2_), and at the respective termination of experiment (192 h for Ascocoryne sp. and Phoma sp.; 216 h for all other tested fungi) (t_3_).*

In summary, the detection of DBP metabolites presented in Tables 7, 8, and in the Supplementary Table S1 clearly confirms biotransformation of DBP by *Ascocoryne* sp., *Phoma* sp., *S. chlorohalonata*, *S. rugosoannulata*, and *T. porosum*. A corresponding compilation of major biochemical steps proposed for the metabolization of DBP by these fungi based upon a combined consideration of the respective metabolite structure proposals, relative intensities of metabolite peak areas, and time courses of both DBP removal and metabolite formation in conjunction with corresponding PB effects (Figure 2, Tables 2, 7, 8, and Supplementary Table S1), is presented in Figure 4. Potentially, the addition of methanol to fungal cultures (applied to improve DBP solubility) may have resulted in overestimations of methylated DBP derivatives presumably formed by transesterification. Fungal, as well as bacterial, cutinases, esterases, and lipases are well known to utilize short-chain alcohols such as methanol as substrates in transesterification reactions (Kim et al., 2005; Okamoto et al., 2011; Lotti et al., 2015). However, such possible experimental artifacts would not have impaired the identification of most of the major reaction steps indicated by blue solid arrows in Figure 4.

## Discussion

In this study the ability of ecophysiologically diverse asco- and basidiomycete fungi to biotransform the PE representatives DBP and DEP, and the plastic precursor chemical BPA was demonstrated. UPLC-QTOF-MS analysis of DBP metabolites in fungal cultures confirmed DBP biotransformation and enabled us to establish the major biochemical reaction steps contributing to DBP metabolization in the Baltic Sea isolate *Ascocoryne* sp., the freshwater-derived strain *Phoma* sp., the environmentally ubiquitous mold *S. chlorohalonata* (all belonging to the Ascomycota), the litter decaying basidiomycete *S. rugosoannulata* and the basidiomycetous yeast *T. porosum* (Figure 4).

In *Ascocoryne* sp., concomitant initial hydrolytic de-esterification and cytochrome P450-catalyzed monohydroxylation of DBP at similar rates is suggested. These initial steps are supported by comparatively high relative intensities of the corresponding transformation products (MBP and TP 317, respectively), the missing influence of PB on MBP formation, and its clearly suppressive effect on the formation of TP 317 (Figure 4 and Tables 7, 8). *Ascocoryne* sp. biotransformed DBP to a greater degree than DEP (Figure 3 and Table 3), despite DBP having a lower aqueous solubility (log *K*_ow_ = 4.27 vs. log *K*_ow_ = 2.54; Cousins and Mackay, 2000; Net et al., 2015) and hence presumably lower bioavailability compared to DEP. This suggests that neither potential bioavailability limitations caused by compound solubility, nor the greater carbon chain lengths of the alkyl esters had a negative influence on the efficiency of the initial PE biotransformation step(s) in this fungus. Similarly, a slightly more efficient hydrolysis of DBP than of DEP was reported for a bacterial PE-hydrolyzing enzyme isolated from culture broth of *Nocardia erythropolis* (Kurane et al., 1984). Hydrolysis of structurally related PEs, including DBP and DEP, at similar rates has also been reported for other bacteria and isolated esterase preparations (both bacterial and eukaryotic, i.e. from bovine pancreas), whereas PEs with branched and hence more bulky alkyl substituents were comparatively hydrolyzed slower (Saito et al., 2010; Huang et al., 2019). Furthermore, extracellular esterases have been implicated in initial PE hydrolysis in fungi (Lee et al., 2007; Hwang et al., 2012; Ahuactzin-Perez et al., 2016). A substantial role of hydrolytic PE de-esterification in *Ascocoryne* sp. as stipulated above is in line with the aforementioned observations. In this context, an initial attack on PEs by extracellular enzymes could potentially help to avoid or diminish bioavailability limitations related to compound uptake by fungal cells for intracellular biotransformation steps.

By contrast, a more efficient DEP biotransformation compared to that of DBP, as observed with all other fungi investigated in this respect (Figure 3 and Tables 4–6), may indicate a possible influence of PE bioavailability on the biotransformation efficiency, as would be expected from the need for compound uptake prior to initial intracellular biotransformation steps. An initial attack on PEs catalyzed by intracellular cytochrome P450s, as implicated in *Phoma* sp. and supported by inhibitory effects of PB on fungal DBP removal (Figure 2 and Table 2), is in accordance with such possible bioavailability effects. The DBP transformation product profile observed with *Phoma* sp. (Tables 7, 8) suggests initial DBP biotransformation dominated by de-esterification via cytochrome P450-catalyzed oxidative *O*-dealkylation, albeit obviously accompanied by DBP monohydroxylation and further oxidation steps to remarkable extents. Cytochrome P450 monooxygenase reactions are previously implicated in the metabolization of the anti-inflammatory pharmaceutical diclofenac by *Phoma* sp. (Hofmann and Schlosser, 2016).

DBP de-esterification to MBP, and subsequent de- and/or transesterification steps controlled by cytochrome P450-dependent initial oxidative dealkylation form the major DBP metabolization pathway in *T. porosum*, whereas DBP hydroxylation and further oxidation of corresponding metabolites seems negligible (Tables 7, 8). This yeast species was described to be inactive on the comparatively more hydrophobic and hence less water soluble PE dioctyl phthalate (DOP; log *K*_ow_ = 7.32; Cousins and Mackay, 2000; Net et al., 2015; Sabev et al., 2006) possibly due to a lower bioavailability and/or mechanistic biochemical constraints of the degradability of DOP compared to DBP. Limited growth of *Trichosporon* DMI-5-1, a yeast isolate obtained from costal sediment, on different DMPEs was reported previously (Luo et al., 2011). Initial hydrolysis of DMPEs by *Trichosporon* DMI-5-1 led to the accumulation of aromatic products (monoesters, terephthalic acid), without their further breakdown.

Prominent initial bioconversion of DBP to MBP via oxidative O-dealkylation, and subsequent production of TP 217, TP 203, and PA, governed by cytochrome P450-dependent reactions is also suggested for both *S. chlorohalonata* and *S. rugosoannulata* (Tables 7, 8), although these fungi are phylogenetically distant. Contrary to *T. porosum*, cytochrome P450-dependent hydroxylation reactions can be deduced to appreciable extents for *S. chlorohalonata* and *S. rugosoannulata*.

The present study establishes a prominent role of cytochrome P450 monooxygenase-catalyzed reactions during DBP metabolism in the majority of the investigated fungi, with corresponding results remaining inconclusive only for the peatland isolate *Acephala* sp. and the Baltic Sea isolate *P. arenariae* (see sub-section Fungal DBP Removal and Influence of PB). DBP hydroxylation and oxidative *O*-dealkylation processes, as stipulated for the investigated fungi above and compiled in Figure 4, represent typical reactions of cytochrome P450s also relevant to mammal PE metabolism (Choi et al., 2012, 2013; Girvan and Munro, 2016; Cantú Reinhard and De Visser, 2017). Cytochrome P450-catalyzed oxidations are commonly substrate-specific, as well as regio-, and frequently stereoselective, with different isoenzymes being responsible for the respective type of reaction, its selectivity, and hence also product variability (Choi et al., 2012; Syed and Yadav, 2012; Girvan and Munro, 2016; Cantú Reinhard and De Visser, 2017). The enormous catabolic versatility of certain fungal lineages is partly based on corresponding inventories of multiple cytochrome P450-encoding genes (Harms et al., 2011; Syed and Yadav, 2012; Syed et al., 2013). The occurrence of isomeric DBP biotransformation products observed within the present study (Supplementary Table S1) has also been reported for human di-2-ethylhexyl terephthalate metabolism (Silva et al., 2015), and may well reflect superimpositions of reactions of P450 isoenzymes with different selectivities. The influence of cytochrome P450-dependent reactions on the formation of the potential TP 203 precursor TP 217 observed with *S. chlorohalonata*, *S. rugosoannulata*, and *T. porosum* (Table 7) may be explained by the cytochrome P450-catalyzed formation of an upstream intermediate, since TP 217 most likely directly arises from a transesterification and not from a cytochrome P450 reaction. The same may apply to TP 203 and PA, where an influence of cytochrome P450-dependent steps on compound formation by the aforementioned fungi was also observed (Table 7). However, TP 203 and PA could also be produced from TPs 217 and 203, respectively, by cytochrome P450-dependent *O*-demethylation reactions (Girvan and Munro, 2016). *O*-demethylation reactions are well-known from fungi, where cytochrome P450 lanosterol 14α-demethylase is a prominent target for antifungal agents (Durairaj et al., 2016; Wang et al., 2019), and also from bacteria (Mallinson et al., 2018). The successive demethylation of TP 217 (dimethyl phthalate ester) via TP 203 (monomethyl phthalate ester) to PA was proposed for complex microbial communities of soil (Cartwright et al., 2000).

Different from *S. chlorohalonata*, *S. rugosoannulata*, and *T. porosum*, cytochrome P450-dependent reactions did not control the production of TP 217 and PA by *Ascocoryne* sp. and *Phoma* sp. (Figure 4 and Table 7). In these fungi, transesterification and hydrolytic de-esterification reactions apparently govern the formation of TP 217 and PA, respectively. Hereby, PA could be produced from DBP in two successive hydrolytic steps, with MBP being formed as an intermediate. PA production by DEHP hydrolysis via the corresponding monoester has been proposed for the white-rot basidiomycete *Pleurotus ostreatus* (Ahuactzin-Perez et al., 2018). Rapid complete enzymatic hydrolysis of various PEs followed by spontaneous oxo-bridge formation to yield 1,3-isobenzofurandione (IBF) was reported for fungal cutinase, whereas application of fungal esterase to the same PEs yielded the corresponding monoesters in addition to IBF (Kim et al., 2003, 2005; Kim and Lee, 2005; Kim and Song, 2009). A bacterial esterase converting structurally diverse PEs into their corresponding monoesters, and a monoester hydrolase subsequently producing PA from such monoesters have also been described (Huang et al., 2019). PA can additionally be formed by hydrolytic demethylation steps from products of transesterification reactions such as TPs 217 and 203 (Figure 4). Dimethyl phthalate (i.e. TP 217 in Figure 4) hydrolysis to monomethyl phthalate (TP 203) and its subsequent hydrolysis to PA is known from the aforementioned bacterial esterase and monoester hydrolase, respectively (Huang et al., 2019). Alternatively, a novel bacterial feruloyl esterase has been reported to convert DBP, DEP, and dimethyl phthalate (TP 217) directly into PA via hydrolysis steps (Wu et al., 2019).

The transesterification reactions proposed within the present study (Figure 4) corroborate previous research addressing microbial PE metabolism (Cartwright et al., 2000; Kim et al., 2005; Lee et al., 2007; Okamoto et al., 2011). Nevertheless, as an alternative to transesterifications catalyzed by hydrolytic enzymes, methylated products such as those indicated in Figure 4 (TPs 291, 275, 247, 217, 203) could also be produced upon methylation of hydroxyl groups by fungal *O*-methyltransferases; a very common reaction in fungal metabolism of xenobiotics (Harms et al., 2011; Wang et al., 2014; Hofmann and Schlosser, 2016). Enzymatic transesterifications only proceed in the presence of alcohol, which is often used as a solvent for PEs in corresponding studies (Kim et al., 2003, 2005; Kim and Lee, 2005; Okamoto et al., 2011) and thus may bias corresponding results. The environmental relevance of such reactions remains to be established as environmental concentrations of alcohols is expectedly rather low (Okamoto et al., 2011).

It remains to be elucidated whether the central PE intermediate PA is further metabolized by the fungi investigated within the present study (Figure 4). At a first glance, the comparatively low relative intensities of this compound shown in Table 7 do not indicate its accumulation. The breakdown of the aromatic PA core of DEHP to butanediol by *Fusarium culmorum*, and complete degradation of PA by the white-rot fungus *Pleurotus ostreatus* were previously suggested (Ahuactzin-Perez et al., 2016; Ahuactzin-Perez et al., 2018). By contrast, monoaromatic breakdown products of DMPEs were not further degraded by *Fusarium* sp. and yeast (Luo et al., 2011, 2012). The bacterial utilization of non-toxic fungal DBP breakdown products as sole carbon and energy sources has been demonstrated before (Ahuactzin-Perez et al., 2014).

The considerably higher biotransformation rate of BPA observed with *Phoma* sp. compared to *P. arenariae* in the present study (Figure 3 and Tables 4, 6) could be attributed to BPA oxidation by extracellular laccases and/or peroxidases, for which activities were only recorded in *Phoma* sp., in addition to cell-bound enzymes (Cajthaml, 2015; Hofmann and Schlosser, 2016). The demonstrated concomitant biotransformation of more than one organic pollutant observed with several fungi (Figure 3 and Tables 3–6) is a typical fungal characteristic (Harms et al., 2011). To the best of our knowledge, bioconversion of BPA as suggested for *P. arenariae* (Figure 3 and Table 4) has not yet been reported. *Phoma* sp. was already previously shown to act on BPA as well as on other prominent micropollutants present in water (Hofmann and Schlosser, 2016). Moreover, *S. rugosoannulata* has been reported to metabolize BPA in addition to other organic environmental pollutants before (Kabiersch et al., 2011; Pozdnyakova et al., 2018).

The observed degree of micropollutant biosportion onto fungal mycelia recorded within the present study (DBP > BPA > DEP; Figure 3 and Tables 3–6) reflects the hydrophobicity of the tested compounds in terms of their respective octanol-water partition coefficients (log *K*_ow_ values of 4.27, 3.32, and 2.54 for DBP, BPA, and DEP, respectively; Cousins and Mackay, 2000; Margot et al., 2013; Net et al., 2015). Individual differences observed with regard to the biosorptive capacities of the tested fungi (Figures 2, 3 and Tables 2–6) can be attributed to their individual cell surface properties, which may further change in response to growth conditions and proximity of hydrophobic environmental pollutants (Linder et al., 2005; Chau et al., 2009; Hanano et al., 2015). In addition to simple physical removal, biosorption has been implicated in aiding the efficiency and variety of micropollutants actively removed. Nguyen et al. (2014) reported increased biotransformation for a number of compounds via whole-cell treatment, compared to cell-free enzymatic processes. The authors argued that increased exposure to mycelium-associated/intracellular biocatalysts provided access to further catabolic processes in addition to those of extracellular secretions (compound specific). High biotransformation rates have been reported for compounds exhibiting strong sorption to fungal cell surfaces (Hofmann and Schlosser, 2016). One could argue that a local increase in compound concentration (i.e. association with mycelium) would establish a concentration gradient and thereby drive contaminant fluxes toward nearby biocatalysts (Johnsen et al., 2005; Semple et al., 2007). In the present study DBP showed strongest sorption, however it was not always the most efficiently biotransformed micropollutant (Figure 3 and Tables 3–6). Aside from other contributory effects (e.g. enzyme specificities, redox potentials, compound toxicity), it may also be postulated that strong biosorption impedes enzyme access to such compounds. In line with this argument, application of differently hydrophobic PEs to the flagellated protist *Karenia brevis* (the cause of the Florida red tide) resulted in an increase in bioaccumulation and toxicity, and a decrease in PE biodegradation with increasing PE hydrophobicity (Sun et al., 2019).

The results of the present study imply an environmentally ubiquitous fungal potential for the biocatalytic breakdown of plastic additives, which comprises ecophysiologically and phylogenetically diverse filamentous and yeast-like fungi dwelling in marine, freshwater and terrestrial habitats. Being part of the complex microbial communities of terrestrial as well as aquatic environments, such fungal activities may contribute to diminish concentrations and mitigate the adverse effects of PEs and other plastic additives following their release into the environment. The loss of plasticizers from polymers due to biocatalytic process proceeding on polymer surfaces (e.g. biodegradation by fungi-containing biofilms) may make plastics more brittle thus potentially contributing to polymer disaggregation and microplastics formation (Krueger et al., 2015a; Hahladakis et al., 2018). Potential adverse or positive environmental and human health impacts possibly resulting from such processes still need to be assessed (Krueger et al., 2015a; Hahladakis et al., 2018; Jiang, 2018). Finally, fungi capable of attacking plastic additives and other micropollutants could be exploited in biotechnologies aimed at the reduction of environmental contamination from sources such as wastewater treatment plants (Pezzella et al., 2017; Hofmann et al., 2018).

## Data Availability Statement

The raw data supporting the conclusions of this article will be made available on request by the authors, without undue reservation, to any qualified researcher.

## Author Contributions

LC, AC, and DS conceived and designed the experiments. LC and AC performed the experiments. BS performed the UPLC-QTOF-MS analyses. LC, AC, BS, and DS analyzed the obtained data, interpreted the obtained results, and wrote the manuscript.

## Conflict of Interest

The authors declare that the research was conducted in the absence of any commercial or financial relationships that could be construed as a potential conflict of interest.
