# Supplementary material for: Biotransformation of Phthalate Plasticizers and Bisphenol A by Marine-Derived, Freshwater, and Terrestrial Fungi
Source: Front Microbiol. 2020 Feb 28;11:317. doi: 10.3389/fmicb.2020.00317 (PMC7059612; doi:10.3389/fmicb.2020.00317)
Supplement: Supplementary file 1 [file Data_Sheet_1.PDF]

Supplementary Material

Ultra performance liquid chromatography-quadrupole time-of-flight mass spectrometry (UPLC-QTOF-MS) analyses of DBP biotransformation products

The method applied for DBP metabolite analysis was adapted from Jahangiri et al. (2017, 2018). Analysis with high mass resolution mass spectrometry was based on a Waters Acquity^{TM} UPLC system connected to a XEVO XS QTOF-mass spectrometer equipped with an electrospray ionization source (Waters, Eschborn, Germany). Separation of analytes was achieved using an Aquity^{TM} HSS T3 column (100 x 2.1 mm, particle size 1.7 µm; Waters), at a column temperature set to 45°C. Eluent A consisted of deionized water (Q-Gard 2, Millipore, Schwalbach, Germany) and eluent B of methanol, both acidified with 0.1% formic acid. The following elution profile was applied: isocratic elution at 2% B for 0.25 min, linear increase to 99.9% B until 12.25 min, isocratic elution at 99.9% B until 15.0 min, linear decrease to 2% B until 15.1 min, and isocratic elution at 2% B until 17 min. A flow rate of 450 µL/min was applied, and 10 µL of each sample were injected for analysis. Ionization source conditions were as follows: the capillary voltage was set to 0.7 kV (140°C operation temperature). The sampling cone voltage was set to 20 V, source offset at 50 V. Nitrogen and argon were used as cone and collision gases. The desolvation temperature was 550 °C and the gas flow 950 L/h. To ensure accuracy during MS analysis, leucine enkephalin was infused via the reference probe as the lockspray. MS data were collected from m/z 50 to m/z 1200 in negative and positive centroid mode with a 0.15 s scan time. Two sets of data were collected in parallel, using MSE acquisition. One dataset contained low-collision energy data (4 eV, MS; effectively the accurate mass of precursors) and the second dataset elevated-collision-energy data (15-35 eV, MSE; fragmentation mode). High resolution data were processed with MassLynx 4.1 software (Waters). A mass resolution of 20000 was applied with a mass precision of approximately 5 ppm. The identification was done by non-target screening for transformation products by using multivariate statistics by MarkerLynx, and the peak areas were integrated by TargetLynx. Tentative chemical structures of DBP metabolites were proposed upon detected masses, interpretation of fragmentation, and favorable interactions.

References

Jahangiri, E., Seiwert, B., Reemtsma, T., and Schlosser, D. (2017). Laccase- and electrochemically mediated conversion of triclosan: Metabolite formation and influence on antibacterial activity. *Chemosphere* 168**,** 549-558. doi: 10.1016/j.chemosphere.2016.11.030

Jahangiri, E., Thomas, I., Schulze, A., Seiwert, B., Cabana, H., and Schlosser, D. (2018). Characterisation of electron beam irradiation-immobilised laccase for application in wastewater treatment. *Sci Total Environ* 624**,** 309-322. doi: 10.1016/j.scitotenv.2017.12.127

SUPPLEMENTARY TABLE S1 | Proposed structures and further characteristics of DBP biotransformation products detected in fungal cultures by UPLC-QTOF-MS (positive electrospray ionization mode).

**Compound**	**Proposed structure(s)**	**Experimental exact mass $[M+H]^+$ / $[M+Na]^+$**	**Assigned elemental composition**	**Retention time(s) (min)**	**Fragments**	**Detected in**
**Dibutyl phthalate (DBP)**	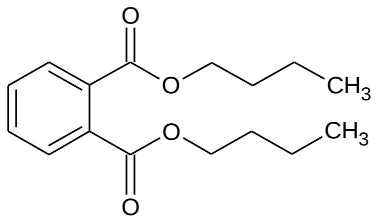	301.143	$C_{16}H_{22}O_4Na$	11.39	133.029 ($C_8H_5O_2$); 149.0235 ($C_8H_5O_3$); 163.038 ($C_9H_7O_3$)	All tested fungi
**Monobutyl phthalate (MBP)** DBP -C_4H_8	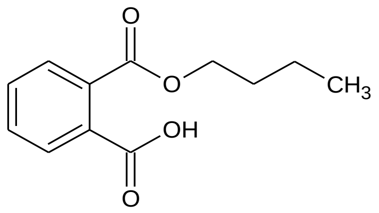	245.08	$C_{12}H_{14}O_4Na$	8.85	105.0337 (C_7H_5O); 133.029 ($C_8H_5O_2$); 163.038 ($C_9H_7O_3$)	All tested fungi
**TP 217** DBP -2x(C_4H_8) +2x(CH_2)	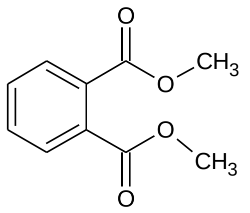	217.048	$C_{10}H_{10}O_4Na$	7.33	133.029 ($C_8H_5O_2$); 163.038 ($C_9H_7O_3$)	All tested fungi
**TP 203** DBP -2x(C_4H_8) +CH_2	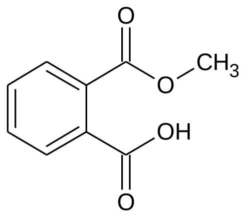	203.032	$C_9H_8O_4Na$	6.00	133.029 ($C_8H_5O_2$); 149.0230 ($C_8H_5O_3$); 163.04 ($C_9H_7O_3$)	All tested fungi
**Phthalic acid (PA)** DBP -2x(C_4H_8)	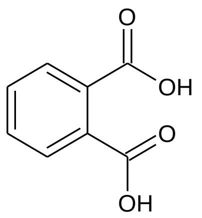	163.04	$C_8H_6O_4$	4.55	Low abundance	All tested fungi

**Compound**	**Proposed structure(s)**	**Experimental exact mass $[M+H]^+$ / $[M+Na]^+$**	**Assigned elemental composition**	**Retention time(s) (min)**	**Fragments**	**Detected in**
**TP 317**<br>DBP +O	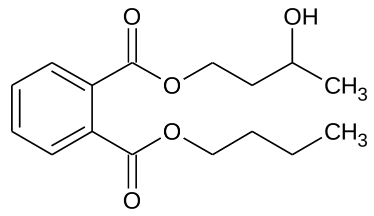<br>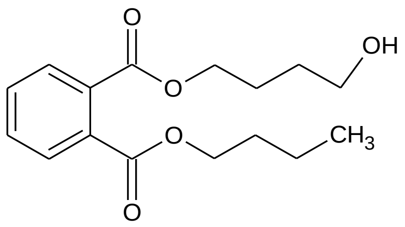<br>Two possible structures shown	317.139	$C_{16}H_{22}O_5Na$	9.79; 9.97	133.029 ($C_8H_5O_2$);<br>149.0235 ($C_8H_5O_3$);<br>163.038 ($C_9H_7O_3$)	*Ascocoryne* sp.;<br>*Phoma* sp.;<br>*S. chlorohalonata*
**TP 315**<br>DBP +O -2H	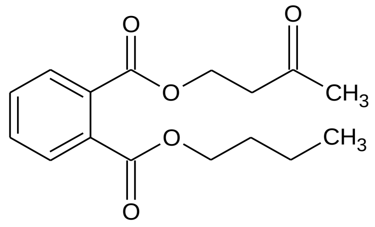	315.121	$C_{16}H_{20}O_5Na$	8.79	133.029 ($C_8H_5O_2$);<br>163.038 ($C_9H_7O_3$)	All tested fungi except *T. porosum*
**TP 275**<br>DBP $-C_4H_8$ $+CH_2$ +O	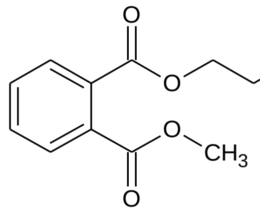	275.089	$C_{13}H_{16}O_5Na$	7.73; 7.89; 7.95	133.029 ($C_8H_5O_2$);<br>163.038 ($C_9H_7O_3$)	All tested fungi
**TP 261**<br>DBP $-C_4H_8$ +O	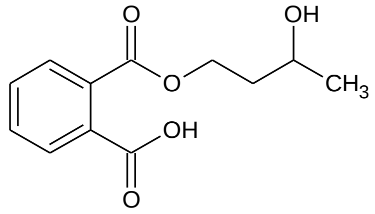<br>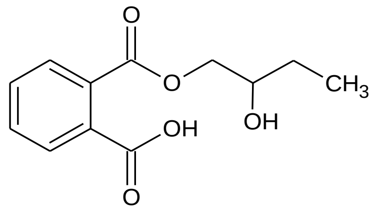<br>Two possible structures shown	261.075	$C_{12}H_{14}O_5Na$	6.73; 6.55	113.0570 (C4H10O2Na);<br>121.0291 (C7H5O2);<br>133.029 ($C_8H_5O_2$);<br>135.0405 (C6H8O2Na);<br>149.0229 (C8H5O3);<br>163.038 ($C_9H_7O_3$)	All tested fungi except *T. porosum*
**TP 259**<br>DBP $-C_4H_8$ +O -2H	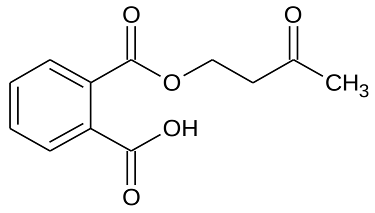	259.059	$C_{12}H_{12}O_5Na$	6.26	163.04 ($C_9H_7O_3$)	*Ascocoryne* sp.;<br>*Phoma* sp.;<br>*S. rugosoannulata*

Compound	Proposed structure(s)	Experimental exact mass $[M+H]^+$ / $[M+Na]^+$	Assigned elemental composition	Retention time(s) (min)	Fragments	Detected in
**TP 247**<br>DBP -2x(C_4H_8) +C_2H_4 +CH_2 +O	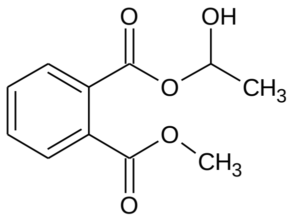	247.059	$C_{11}H_{12}O_5Na$	6.13	120.0212 ($C_7H_4O_2$);<br>133.029 ($C_8H_5O_2$);<br>163.038 ($C_9H_7O_3$);<br>207.0651($C_{11}H_{11}O_4$)	*Phoma* sp.;<br>*S. rugosoannulata*
**TP 333**<br>DBP +2O	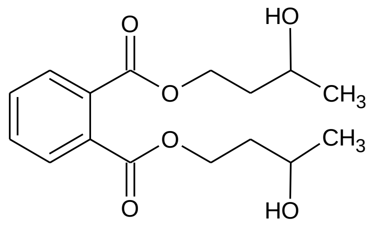	333.141	$C_{16}H_{22}O_6Na$	7.96; 8.07; 8.14; 8.18	133.029 ($C_8H_5O_2$);<br>143.0235($C_8H_5O_3$);<br>163.038 ($C_9H_7O_3$)	All tested fungi except *T. porosum*
**TP 331**<br>DBP +2O -2H	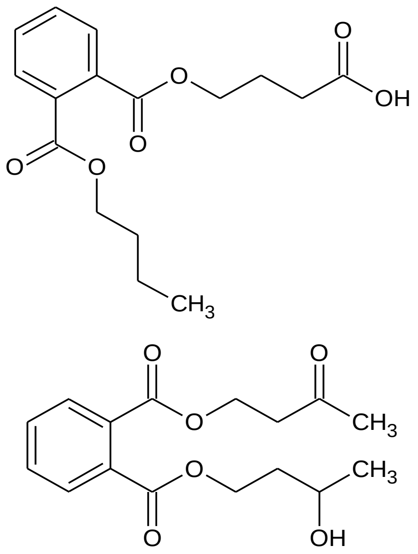<br>Two possible structures shown	331.116	$C_{16}H_{20}O_6Na$	7.62; 9.52	267.061 ($C_{12}H_{13}O_4Na_2$) (9.52 min only);<br>261.074 ($C_{12}H_{14}O_5Na$);<br>163.038 ($C_7H_8O_3Na$) (both 7.62 and 9.52 min)	All tested fungi
**TP 305**<br>DBP -C_2H_4 +2O	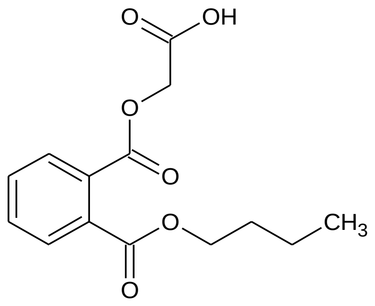	305.101	$C_{14}H_{18}O_6Na$	6.78; 6.90	163.04 ($C_9H_7O_3$)	*Phoma* sp.;<br>*S. rugosoannulata*
**TP 291**<br>DBP -C_4H_8 +CH_2 +2O -2H	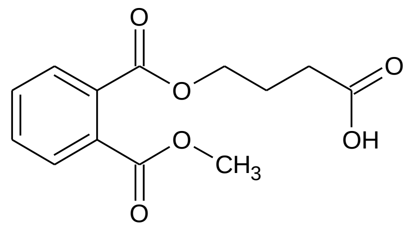	291.085	$C_{13}H_{16}O_6Na$	6.19; 6.35; 6.45; 6.55	Low abundance	*Phoma* sp.;<br>*S. chlorohalonata*;<br>*S. rugosoannulata*

DBP biotransformation products were first grouped together according to the number of additional oxygen atoms (i.e. one or two) in their structures. Within the resulting two groups, metabolites were then sorted in descending order according to their molecular masses. DBP metabolites occurring in the form of different isomers can be identified by the corresponding isomer retention times.
